# Low-calcium diets increase duodenal mRNA expression of calcium and phosphorus transporters and claudins but compromise growth performance irrespective of microbial phytase inclusion in broilers

**DOI:** 10.1016/j.psj.2021.101488

**Published:** 2021-09-16

**Authors:** Y.X. Hu, J. van Harn, W.H. Hendriks, J. van Baal, M.A. Dijkslag, M.M. van Krimpen, P. Bikker

**Affiliations:** ⁎ Wageningen University & Research, Wageningen Livestock Research, Wageningen, 6700 AH, the Netherlands; † Wageningen University & Research, Animal Nutrition Group, Wageningen, 6700 AH, the Netherlands; ‡ ForFarmers, Lochem, 7241 CW, the Netherlands

**Keywords:** Ca and P digestibility, intestinal gene expression, paracellular pathway, transcellular pathway, broilers

## Abstract

The hypothesis that dietary inclusion of microbial phytase improves apparent calcium (**Ca**) digestibility thereby allowing a lower dietary Ca inclusion without compromising growth performance was tested. One-day-old male Ross 308 broilers (25 birds/pen, 9 pens/treatment) were assigned to 8 experimental diets containing one of 4 dietary Ca to retainable P (**rP**) ratios (1.3, 1.8, 2.3, and 2.8) with (1,000 FTU/kg) or without microbial phytase. On d 21 to 23, digesta from different intestinal segments of 8 birds per pen were collected to determine apparent Ca and P digestibility. Mid duodenal mucosa was collected for expression of Ca (CaBP-D28k, PMCA1) and P (NaPi-IIb, PiT-1, PiT-2, and XPR1) transporters by RT-qPCR. Dietary phytase inclusion in low Ca/rP diets increased Ca digestibility in the distal ileum (*P*_interaction_ = 0.023) but not the proximal or distal jejunum. Broilers receiving the lowest Ca/rP displayed the lowest body weight gain, highest feed conversion ratio (*P* < 0.001), and lowest tibia strength, irrespective of dietary phytase inclusion. Incremental dietary Ca/rP linearly reduced P digestibility to a greater extent in the absence of phytase in the distal jejunum and ileum (*P*_interaction_ = 0.021 and 0.001, respectively). Incremental dietary Ca/rP linearly reduced serum P more in phytase-free diets (*P*_interaction_ < 0.001), and lowered duodenal expression of P transporters NaPi-IIb, PiT-2, and XPR1 (*P* = 0.052, 0.071 and 0.028, respectively). Incremental dietary Ca/rP linearly increased (*P* < 0.001) serum Ca irrespective of phytase inclusion, accompanied by a lower (*P* < 0.001) duodenal expression of Ca transporters CaSR, CaBP-D28k and PMCA1 and Ca-pore forming claudins CLDN-2 and CLDN-12. Dietary phytase increased (*P* = 0.026) NaPi-IIb but reduced (*P* = 0.029) CLDN-2 expression. Incremental Ca/rP reduced Ca and P digestibility, increased serum Ca, lowered serum P and inhibited mRNA levels of Ca and P-related transporters, indicating that these transporters and CLDN contribute to the observed effect of dietary Ca and phytase on Ca and P absorption. Despite the improvement in Ca digestibility, dietary phytase did not restore the compromised growth performance and tibia strength of broilers fed a Ca-deficient diet, leading to rejection of the hypothesis.

## INTRODUCTION

Phosphorus (**P**) is the third most expensive ingredient in farm animal diets (Woyengo and Nyachoti, 2011) and plays an important role in many biological processes such as muscle contraction, energy production, cell signaling, and bone formation (Chang and Anderson, 2017). Inositol phosphate bound P (**IP-P**) is the major form of P storage (60–80%) in most cereal grains and oil seeds (Rodehutscord et al., 2016). This IP-P is poorly digestible in non-ruminant animals such as pigs and broilers. It is generally accepted that reduction of dietary calcium (**Ca**) content improves IP degradation and IP-P digestion (Sommerfeld et al., 2019). However, over-reduction of dietary Ca content may compromise growth performance and tibia breaking strength (Hu et al., 2020) because a minimum supply of dietary Ca is required to suffice post-absorption P utilization and bone development. Improving dietary Ca digestibility seems to be a promising approach to reduce dietary Ca inclusion without compromising growth performance or bone development in broilers.

Dietary inclusion of microbial phytase is widely practised to improve IP-P digestion in animal diets (Lei et al., 2013). A number of studies reported that microbial phytase also improves dietary Ca digestibility although the results are inconsistent. The mechanism involved may be that dietary phytase liberates Ca ions by degradation of its chelator IP. Ravindran et al. (2020) showed that microbial phytase inclusion (500 and 2,000 FTU/kg) enhanced the true ileal Ca digestibility in broilers fed canola-based diets, and also in corn-soybean meal based diets at a dosage of 2,000 but not 500 FTU phytase/kg diet. These findings are largely in line with Majeed et al. (2020) who demonstrated that microbial phytase inclusion (1,000 FTU/kg) significantly increased apparent ileal Ca digestibility from 44 to 68% in diets supplemented with coarse limestone, while it reduced Ca digestibility from 70 to 61% in diets with fine limestone. These findings indicate that dietary phytase may improve Ca digestibility depending on dose of microbial phytase and source of Ca, probably because they affect Ca-IP complexes in the gastrointestinal tract (**GIT**) of broilers. It seems possible that inclusion of microbial phytase improves Ca digestibility and allows a reduction of dietary Ca inclusion without compromising growth performance. Moreover, a reduction in dietary Ca may abate the process of Ca-IP complexation, which in turn, may enhance microbial phytase efficacy in the GIT. The interaction between Ca and microbial phytase on Ca and P digestibility is currently not fully clarified with studies reporting a high dietary Ca content hampering or either enhancing microbial phytase efficacy.

The binding capacity of IP toward Ca decreases drastically at a pH <5 (Dersjant‐Li et al., 2015), indicating that phytase efficacy would be hampered more in the distal than proximal segments of the GIT. However, Ca and P absorption along the intestinal tract in broilers is not fully elucidated. Moreover, a gradual reduction in expression of Ca and P transporters along the small intestine of broilers was observed (Li et al., 2018), which may suggest a lower absorption capacity of Ca and P in the distal compared to the proximal part of the small intestine. The candidate genes involved in intestinal Ca and P absorption in broilers are presumed to be rather similar to those in mammals, due to their relative high degree in protein sequence homology, implicating functional similarity (Proszkowiec-Weglarz and Angel, 2013). Transcellular intestinal absorption of Ca or P starts with the apical uptake into the enterocytes via distinct transporters, diffusion across the cytoplasm and basolateral exit by other transporters (Kiela and Ghishan, 2018). The paracellular mechanism is a passive transport process that occurs across the majority of the intestine and is a linear function of luminal Ca and P concentration and is likely mediated by claudins (**CLDN**), integral structures of the tight junction complex (Tsukita et al., 2019). Recently, it has been demonstrated that Ca absorbed through the paracellular shunt is also significantly regulated (Hashimoto et al., 2020). More insight into the modulation of intestinal Ca and P transporters and CLDN through dietary intervention is an important element in poultry nutrition in order to better understand and improve intestinal Ca and P absorption, reduce feed costs and alleviate P pollution in the environment.

We hypothesized that microbial phytase in the diet would increase intestinal Ca absorption in broilers, allowing lower dietary Ca inclusion without affecting the growth performance. Therefore, we investigated the interactive effect of dietary Ca content and microbial phytase inclusion on the growth performance, apparent Ca and P digestibility, serum Ca and P content as well as duodenal mRNA expression of genes related to Ca and P absorption in broilers.

## MATERIALS AND METHODS

The experiment was approved by the ethical committee of Wageningen University & Research (2016.D-0065.022) and conducted in the facilities of ForFarmers, Bathmen, the Netherlands. All procedures agreed with Dutch laws on animal experiments in accordance with EU directive 2010/63.

### Experimental Design and Diets

A total of 1,800 one-day-old Ross 308 male broilers were equally divided over 72 pens, receiving one of 8 experimental diets containing one of 4 Ca to retainable P ratios (**Ca/rP**) in the presence (1,000 FTU/kg) or absence of microbial phytase in a 4 × 2 completely randomized block design, with 9 replicate pens per treatment and 25 birds per pen. Pens were blocked by location within the barn and allotted to one of 8 experimental treatments. The intended rP content (3.2, 2.5, and 2.2 g/kg, excluding the contribution of microbial phytase, in the starter, grower, and finisher period, respectively) was fixed at 80% of CVB (2018b) recommendation (4.0, 3.1, and 2.8 g/kg for the starter, grower, and finisher broilers, respectively) for all treatment groups. The intended dietary Ca/rP of 1.3, 1.8, 2.3, and 2.8 was realized by inclusion of various amounts of limestone at the expense of diamol according to experimental design.

The animal experiment lasted for 36 d and included 3 feeding phases. Starter, grower, and finisher diets were supplied from d 0–9, 10–28, and 29–36, respectively. In each of the 3 feeding phases, a basal diet, which met or exceeded the minimum requirement of all nutrients for broilers (CVB, 2018b) except for Ca and rP, was prepared and then divided into 8 equal portions. Subsequently, the eight experimental diets were made by adding various amounts of limestone (Faunacal, Wülfrath, Germany; mean particle size 90 µm as reported by the manufacturer), phytase (Axtra Phy, Danisco Animal Nutrition, Marlborough, United Kingdom) and diamol (Damolin, Kønsborgvej, Denmark) according to the experimental design. Titanium dioxide (**TiO_2_**) was added to the basal grower diets at 5 g/kg as an indigestible marker. All diets were pelleted (starter diets 2.5 mm, grower and finisher diets 3.2 mm). Diet samples were taken with an automatic sampling device during production. Dietary composition is reported in Table 1. Intended and analyzed Ca, P, and phytase activity are shown in Table 2.

**Table 1:** Ingredients and composition of the diets, g/kg as fed unless otherwise specified.

Items	Starter	Grower	Finisher
Ingredients			
Wheat	320.0	318.0	350.0
Maize	257.0	260.0	253.0
Soybean meal (48% crude protein)	307.0	278.0	238.0
Rapeseed meal	24.8	39.8	59.1
Sunflower seed meal	5.3	10.0	10.9
Soybean oil	27.6	36.0	38.7
Palm oil	5.0	10.0	10.0
Oat hulls	10.0	10.0	10.0
Lauric fatty acids	5.0	5.0	5.0
Premix vitamins^1^	2.0	1.5	1.25
Premix trace minerals^2^	1.5	1.5	1.25
Premix xylanase (6.25%)^3^	1.0	1.0	1.0
DL-Methionine	2.8	2.3	2.1
L-Lysine (HCl)	2.4	1.7	1.8
L-Threonine	1.0	0.5	0.5
Valine	0.7	0.1	0.1
Sodium bicarbonate	2.3	3.0	2.7
Sodium chloride	1.8	1.0	1.0
Monocalcium phosphate	9.3	5.5	4.4
Experimental mixture^4^	13.5	10.1	9.2
Titanium dioxide	0.0	5.0	0.0
Nutrients, calculated			
AMEn, kcal/kg	2,900	3,000	3,025
Crude protein	219	211	200
Crude fiber	33	34	36
Crude ash	57	50	46
Crude fat	63	77	80
Starch	357	360	372
Nutrients, analyzed			
Dry matter	890	882	881
Crude ash	55	51	43
Crude fiber	32	33	34
Crude protein	216	207	199
Starch	367	355	369
Crude fat	65	75	81

1 Vitamin premix (Trouw Nutrition, Putten, the Netherlands) contained: (IU/kg) vitamin A 5,000,000; vitamin D_3_ 2,222,000; vitamin E 13,333; (mg/kg) vitamin K_3_ 1,000; vitamin B_1_ 667; vitamin B_2_ 2,667; vitamin B_3_ 26,667; vitamin B_5_ 7246; vitamin B_6_ 2,000; vitamin B_7_ 89; vitamin B_11_ 667; vitamin B_12_ 10.

2 Trace mineral premix (Trouw Nutrition, Putten, the Netherlands) contained: (g/kg) FeSO_4_•H_2_O 23.3; Ca(IO_3_)_2_ 1; CuSO_4_•5H_2_O 8; MnO 46.7; ZnSO_4_•H_2_O 53.3; (mg/kg) Na_2_SeO_3_ 133.

3 Xylanase 6.25% (Trouw Nutrition, Putten, the Netherlands).

4 Composition shown in Table 2.

**Table 2:** Inclusion of limestone, phytase, and diamol, and calculated and analyzed content of calcium (Ca), phosphorus (P) and phytase activity in the starter, grower, and finisher diets, g/kg as fed unless otherwise specified.

Phytase, FTU/kg	0				1,000			
Ca/rP	1.3	1.8	2.3	2.8	1.3	1.8	2.3	2.8
Starter period (d 0–10)								
Limestone^1^	0.5	4.9	9.2	13.5	0.5	4.9	9.2	13.5
Phytase^2^	0	0	0	0	0.2	0.2	0.2	0.2
Diamol^3^	13.0	8.6	4.3	0	13.0	8.6	4.3	0
Ca calculated	4.2	5.8	7.4	9.0	4.2	5.8	7.4	9.0
P calculated	5.8	5.8	5.8	5.8	5.8	5.8	5.8	5.8
Retainable P calculated^4^	3.2	3.2	3.2	3.2	3.2	3.2	3.2	3.2
Ca analyzed	4.5	6.0	6.8	9.0	4.5	6.5	8.0	9.0
P analyzed	5.7	5.7	5.6	5.6	5.7	5.6	5.7	5.6
Phytase analyzed, FTU/kg	119	34	404	246	1395	1550	1362	1511
Grower period (d 10–29)								
Limestone^1^	0.06	3.4	6.8	10.1	0.06	3.4	6.8	10.1
Phytase^2^	0	0	0	0	0.2	0.2	0.2	0.2
Diamol^3^	10.1	6.7	3.3	0	10.1	6.7	3.3	0
Ca calculated	3.2	4.5	5.7	6.9	3.2	4.5	5.7	6.9
P calculated	5.0	5.0	5.0	5.0	5.0	5.0	5.0	5.0
Retainable P calculated^4^	2.5	2.5	2.5	2.5	2.5	2.5	2.5	2.5
Ca analyzed	3.5	4.7	6.0	7.1	3.5	4.7	5.9	6.7
P analyzed	4.7	4.8	4.9	4.9	5.1	4.9	5.2	5.0
Phytase analyzed, FTU/kg	237	135	55	293	1386	1060	1205	860
Finisher period (d 29–36)								
Limestone^1^	0.1	3.1	6.2	9.2	0.1	3.1	6.2	9.2
Phytase^2^	0	0	0	0	0.2	0.2	0.2	0.2
Diamol^3^	9.1	6.1	3.0	0	9.1	6.1	3.0	0
Ca calculated	2.9	4.0	5.2	6.3	2.9	4.0	5.2	6.3
P calculated	4.8	4.8	4.8	4.8	4.8	4.8	4.8	4.8
Retainable P calculated^4^	2.2	2.2	2.2	2.2	2.2	2.2	2.2	2.2
Ca analyzed	3.2	4.1	5.5	6.1	3.2	4.2	5.2	6.1
P analyzed	4.8	4.7	4.8	4.8	4.8	4.9	4.7	4.8
Phytase analyzed, FTU/kg	252	315	281	157	966	1208	673	842

1 Faunacal, Wülfrath, Germany, particle size 90 µm as reported by the manufacture.

2 Axtra Phy, Marlborough, United Kingdom.

3 Damolin, Kønsborgvej, Denmark.

4 Calculated retainable P content does not include contribution of microbial phytase (CVB, 2018a).

### Animal Husbandry and Management

The birds were housed in 2 m^2^ pens with wooden shavings (0.9–1.0 kg/m^2^) as bedding material. During the first 3 d continuous light (24L:0D) was provided, thereafter, a dark/light schedule of 18 h light and 6 h dark (18L:6D, 20 lux light intensity) was used throughout the experimental period except for the week prior to dissection (d 17–22) during which continuous light was provided (24L:0D) to ensure a constant feed intake and steady-state passage of digesta in the GIT. The birds were weighed on d 10, at dissection (d 21–23), d 29 and d 36. On d 21 to 23, eight birds per pen were randomly selected and sacrificed for collection of digesta, mucosa scrapings and tibia (see below). The remaining 17 birds per pen were kept until d 36 to determine the growth performance in the overall period (d 0–36) until reaching a commonly used commercial slaughter weight. Broilers were dissected per block with 3 blocks per day on d 21 to 23. From d 18 to 23, a cardboard was placed in the pens to prevent birds from consuming bedding material since this might influence the observed Ca and P digestibility. During the entire period, birds had free access to feed and water. Ventilation and temperature in the barn was computer controlled and were appropriate for the age of birds.

### Sample Collection and Chemical Analysis

On d 21 to 23, eight birds per pen were weighed and killed by electronic stunning and exsanguination. The abdominal cavity was opened, the GIT carefully taken out and laid out on the dissection table, before the proximal and distal half of the jejunum as well as the distal half of the ileum were enclosed with plastic forceps and quantitatively emptied by flushing with deionized water. Digesta in the proximal ileum were not collected since our previous study (Hu et al., 2020) indicated that apparent Ca and P digestibility in the proximal ileum were similar to that in the distal ileum. The collected digesta were stored at −20°C until determination of Ca, P, and Ti. After electronical stunning blood was collected from the carotid artery of 3 of the 8 dissected birds per pen, and centrifuged at 3,000 × *g* for 10 min at 4°C to harvest serum. The serum was stored at −80°C pending analysis of Ca and P. After longitudinally opening the intestinal segments with the use of a pair of scissors and gently washing with a slow stream of tap water to rinse off residual digesta, mucosa was scraped from the mid duodenum (approximately 5 cm) of one bird, randomly selected out of the 8 dissected birds per pen, snap-frozen in liquid nitrogen and stored at −80°C prior to analysis of gene expression. The right tibia from three birds, randomly selected out of the 8 dissected birds per pen was separated and stored at −20°C prior to breaking test.

Fresh diets and lyophilized digesta were ground to pass a 1-mm sieve (Retsch GmbH, Germany) prior to subsequent analyses. Diets were analyzed for dry matter (ISO, 1999b), crude ash (ISO, 2002a), crude fiber (ISO, 2002b), crude protein (N (ISO, 2005) × 6.25), starch (ISO, 2004), and crude fat (ISO, 1999a) before commencement of the trial. The Ca, P, and Ti content in the diets and lyophilized digesta were determined using ICP-OES (ThermoFisher, MA; ISO, 2009) after destruction of the samples with a mixture of 37% HCl (6 mL), 65% HNO_3_ (2 mL), and 48% HF (2 mL) in a microwave (CEM, NC; Wang et al., 2004). Serum Ca and P concentrations were determined with a C701 Photometric measuring unit (Roche Diagnostics Limited, Rotkreuz, Switzerland). The characteristics of tibia strength were determined by a 3-point bending test using an Instron Texture Analyzer (type 3366, MA), as described by Guz et al. (2019). Briefly, the bones were placed underneath a chisel which slowly moved downward and gradually increased pressure to break the bone. The real-time pressure and distance of the chisel were automatically recorded by a computer. Using these data, the tibia breaking strength and stiffness (slope) were determined as characteristics of tibia strength.

Expression of genes in the duodenal mucosa was determined using real-time quantitative polymerase chain reaction (**RT-qPCR**) following the standard protocol in our lab. Briefly, scrapings of duodenal mucosa were ground in liquid nitrogen and subsampled (50–100 mg). In the subsample, total RNA was isolated using TRIzol (ThermoFisher Scientific) and then subjected to on-column DNase treatment to remove possible genomic DNA contamination with the Nucleospin II kit (Macherey Nagel, Düren, Germany). Quantity and quality of RNA was determined with the NanoDrop 1000 Spectrophotometer (ThermoFisher Scientific) and 2100 Bioanalyzer and RNA 6000 Nano LabChip kit (Agilent Technologies, Santa Clara, CA), respectively. The RNA integrity number values ranged from 9.5 to 10. A total of 500 ng RNA was reverse transcribed with Superscript III kit (ThermoFisher Scientific) and mRNA levels were assessed by RT-qPCR amplification on a QuantStudio 5 Real-Time PCR System (ThermoFisher Scientific) using the SensiFAST SYBR low-ROX Kit (Bioline, Alexandria, Australia) under the following conditions: 95°C for 15 s and 60°C for 30 s for 40 cycles. Absolute quantitative mRNA measurement was performed by establishing a linear calibration curve using 10-fold serial dilutions of cDNA template for corresponding genes. Expression levels of gene of interest were normalized to the geometric mean expression level of importin 8 (**IPO8**), elongation factor 2 (**EEF2**), beta actin (**ACTB**), glyceraldehyde 3-phosphate dehydrogenase (**GAPDH**), and 60S ribosomal protein (**RPLP0**). Primer sequences used in RT-qPCR analysis are presented in Table 3.

**Table 3:** Gene-specific primers used for the analysis of mRNA levels using real-time quantitative polymerase chain reaction (RT-qPCR).

Gene	Accession no.	Sense 5′-3′	Antisense 5′-3′
CaSR	XM_416491.6	GCCAATCTGCTGGGACTCTT	CTGATGCTCGTCATTGGGGA
TRPV6	XM_004938143.3	GACCAGAGCAAAGAGGGACC	CCGCCTCTGCATGAGGTATT
TRPC1	NM_001004409.2	GAATACGATGGGACCAGCCC	AGCTGATTGCGTGACTCTTCT
CaBP-D28k	NM_205513.1	CGAGATCTGGCACCACTACG	ACCTGAGCAAGCTCAACGAT
PMCA1	NM_001168002.3	ACTCTGATGGCAGTTTCCGA	GTCACGGTCCCTAGGTCTGA
NaPi-IIa	XM_015293846.2	GAAGCCAGGTGCCTCTGATG	AGAGGATGGCGTTGTCCTTG
NaPi-IIb	NM_204474.2	TGGCTTTGTCCCTGCTTGTT	CCAGCCAGCCAAGTAAAAGG
PiT-1	XM_015297502.2	TGAAGCTTCCCATCTCGGGT	AGGACAACACGATTTTTAGCAGC
PiT-2	NM_001305398.1	GCTGGGAGCAAAAGTAGGAGA	AAACAGCAGAACCAACCATCG
XPR1	XM_422258.6	AACCTGGAGACAACACGAGG	CGTTGGTCACCACTTCCTCT
ZO-1	XM_015278981.2	CCGCAGTCGTTCACGATCT	GGAGAATGTCTGGAATGGTCTGA
CLDN-2	NM_001277622.1	CAACTGGAAGATCAGCTCCT	TGTAGATGTCGCACTGAGTG
CLDN-12	XM_025148431.1	CTCTTATTCCTCCTCGCATG	GTCAAAGCTAAAGACAGGCT
CLDN-16	XM_426702.4	GGGATCCAAACATGTGATGA	AGAGAAATCCAAATCCTGCC
VDR	NM_205098.1	GGCTCAGGTTTTGCAGATTTG	CAGCATCGCCTTTCCCATT
ACTB	NM_205518.1	GCCCTGGCACCTAGCACAAT	GCGGTGGACAATGGAGGGT
EEF2	NM_205368.1	CAGTTGGCTTTGGTTCTGGC	AAAGTATCTGTCTCCCCACAGC
GAPDH	NM_204305.1	ATCCCTGAGCTGAATGGGAAG	AGCAGCCTTCACTACCCTCT
IPO8	XM_015287054.2	ACCTCCGAGCTAGATCCTGT	GGCTCTTCTTCGCCAACTCT
RPLP0	NM_204987.2	TTGGGCATCACCACAAAGATT	CCCACTTTGTCTCCGGTCTTAA

Abbreviations: ACTB, beta actin; CaSR, Ca^2+^ sensing receptor; CaBP-D28k, calbindin D28k; CLDN, claudin; EEF2, eukaryotic translation elongation factor 2; GAPDH, glyceraldehyde 3-phosphate dehydrogenase; NaPi-IIa, sodium dependent phosphate transporter IIa; NaPi-IIb, sodium dependent phosphate transporter IIb; PMCA1, plasma membrane Ca-ATPase 1; IPO8, importin 8; PiT-1, inorganic phosphate transporter 1; PiT-2, inorganic phosphate transporter 2; RPLP0, 60S acidic ribosomal protein P0; VDR, vitamin-D_3_ receptor; TRPV6, transient receptor potential cation channel subfamily V member 6; TRPC1, transient receptor potential canonical 1; XPR1, polytrophic retrovirus receptor 1; ZO-1, zonula occludens-1.

### Calculations and Statistical Analysis

The apparent Ca and P digestibility coefficient was calculated according to de Vries and Gerrits (2018):

$$\text{Apparent}\;\;\text{digestibility},\;\;\%=\left(1-\left(X_{\text{digesta}}/X_{\text{diet}}\right)\times \left(Ti_{\text{diet}}/Ti_{\text{digesta}}\right)\right)\times 100$$

where X_digesta_ and X_diet_ are the Ca or P content in the freeze-dried digesta and diet (g/kg), respectively, and Ti_diet_ and Ti_digesta_ the Ti content in diet and freeze-dried digesta (g/kg), respectively.

Pen was the experimental unit for data analysis. All data were subjected to a two-way ANOVA using the MIXED procedure of SAS (version 9.4, SAS Institute, Cary, NC) with dietary Ca/rP, phytase and their interaction as fixed effects and block and pen as random effects. The LSMEANS procedure with a PDIFF option was used to separate the means. Distribution and variance homogeneity of the Studentized residual were visually checked by the graphics plotted using the ODS GRAPHICS procedure. A CONTRAST procedure was used to estimate the linear and quadratic effect of dietary Ca/rP irrespective of phytase inclusion. Probability was considered significant at *P* ≤ 0.05 and a trend at 0.05 < *P* ≤ 0.10.

## RESULTS

### Growth Performance

The mean initial body weight (**BW**) of the newly hatched broilers (d 0) across dietary treatments was approximately 47 g (Table 4). Intake of microbial phytase (1,000 FTU/kg) increased body weight gain (**BWG**) and feed intake (**FI**) and lowered feed conversion ratio (**FCR**) in the first 3 weeks of life (*P* = 0.002, 0.029, and 0.001, respectively). Furthermore, dietary Ca content quadratically affected BWG, FI, and FCR (*P* < 0.001, 0.004 and < 0.001, respectively), with the maximum values of BWG and FI being observed in broilers fed a dietary Ca/rP of 1.8. This impact of dietary Ca/rP and phytase on growth performance persisted until the end of the trial (d 36). No significant Ca/rP × phytase interactions for BWG, FI, and FCR were observed during the entire experiment (d 0–36).

**Table 4:** Least square means of initial body weight on d 0 (BW d0), body weight gain (BWG), feed intake (FI), and feed conversion ratio (FCR) of broilers from d 0 to dissection (d 21–23) and d 36 as affected by dietary Ca to retainable P ratio (Ca/rP) and microbial phytase supplementation^1^^,^^2^.

			D 0 to dissection (d 21–23)			D 0–36		
Ca/rP	Phytase, FTU/kg	BW d0, g	BWG, g	FI, g	FCR, g/g	BWG, g	FI, g	FCR, g/g
1.3	0	47.2	1,132	1488	1.32	2,483	3,790	1.53
1.8	0	46.9	1,190	1518	1.28	2,661	3,897	1.46
2.3	0	46.8	1,178	1497	1.27	2,656	3,868	1.46
2.8	0	46.9	1,125	1451	1.29	2,609	3,803	1.46
1.3	1,000	47.1	1,138	1476	1.30	2,511	3,781	1.51
1.8	1,000	46.7	1,233	1552	1.26	2,734	3,968	1.45
2.3	1,000	47.0	1,204	1526	1.27	2,667	3,888	1.46
2.8	1,000	46.9	1,204	1518	1.26	2,699	3,901	1.45
SEM		0.35	23.8	26.5	0.010	39.9	52.2	0.011
Ca/rP								
1.3		47.2	1,135^c^	1,482^b^	1.31^a^	2,497^b^	3785^c^	1.52^a^
1.8		46.8	1,211^a^	1,535^a^	1.27^b^	2,697^a^	3932^a^	1.46^b^
2.3		46.9	1,191^ab^	1,511^ab^	1.27^b^	2,661^a^	3878^ab^	1.46^b^
2.8		46.9	1,165^bc^	1,484^b^	1.28^b^	2,654^a^	3,852^bc^	1.46^b^
SEM		0.24	16.9	18.7	0.007	27.8	37.7	0.008
Phytase								
0		47.0	1,156	1,488	1.29	2,602	3,839	1.48
1000		46.9	1,195	1,518	1.27	2,653	3,884	1.47
SEM		0.17	11.9	13.2	0.005	19.6	37.5	0.006
*P*-value								
Ca/rP		0.491	<0.001	0.019	<0.001	<0.001	0.003	<0.001
Linear		-	0.197	0.800	<0.001	<0.001	0.340	<0.001
Quadratic		-	<0.001	0.004	<0.001	<0.001	0.001	<0.001
Phytase		0.845	0.002	0.029	0.001	0.010	0.073	0.057
Ca/rP × Phytase		0.878	0.186	0.235	0.286	0.367	0.393	0.585

a,b,c Values within a column without common superscript differ significantly (*P* ≤ 0.05).

1 Calculated rP content was 3.2, 2.5, and 2.2 g/kg in the starter, grower and finisher diets, respectively, excluding the contribution of microbial phytase (CVB, 2018a).

2 Nine replicate pens per treatment (n = 9).

### Apparent Digestibility in Different Intestinal Segments and Serum Concentration of Ca and P

Overall, apparent Ca digestibility gradually increased from the proximal jejunum to distal ileum (Table 5). Incremental dietary Ca/rP linearly reduced (*P* < 0.001) apparent Ca digestibility in all 3 intestinal segments. Phytase inclusion did not affect apparent Ca digestibility in the jejunum, whereas in the distal ileum, we observed a Ca/rP × phytase interaction (*P*_interaction_ = 0.023). In particular, addition of phytase enhanced apparent Ca digestibility only in the 1.3 and 1.8 Ca/rP diets compared to the non–phytase-treated broilers. Despite its inhibitory action on apparent Ca digestibility, incremental dietary Ca/rP, but not phytase inclusion (*P =* 0.296), linearly and quadratically elevated (*P ≤* 0.001) serum Ca concentration.

**Table 5:** Least square means of calcium (Ca) and phosphorus (P) content in the serum and apparent Ca and P digestibility in different gastrointestinal tract segments on d 21–23 in broilers, in response to dietary Ca to retainable P ratio (Ca/rP) and microbial phytase supplementation^1^^,^^2^.

		Serum, mM		Apparent Ca digestibility, %			Apparent P digestibility, %		
Ca/rP	Phytase, FTU/kg	Ca	P	Proximal jejunum	Distal jejunum	Distal ileum	Proximal jejunum	Distal jejunum	Distal ileum
1.3	0	2.43	2.31^a^	46.1	56.1	67.3^bc^	61.2	73.1^b^	78.8^b^
1.8	0	2.64	2.14^c^	43.6	54.2	63.2^c^	49.2	62.0^c^	65.3^e^
2.3	0	2.68	1.97^d^	37.1	47.1	54.2^d^	43.9	54.6^d^	56.2^f^
2.8	0	2.74	1.72^e^	27.4	40.4	48.9^e^	32.3	46.2^e^	49.3^g^
1.3	1,000	2.43	2.34^a^	55.8	63.4	75.6^a^	76.0	84.3^a^	89.4^a^
1.8	1,000	2.62	2.25^ab^	51.5	59.0	69.6^b^	72.6	82.3^a^	83.7^a^
2.3	1,000	2.64	2.17^bc^	33.8	45.7	54.8^d^	59.8	72.9^b^	75.2^c^
2.8	1,000	2.71	2.17^bc^	27.4	38.2	46.9^e^	57.3	68.5^b^	70.3^d^
SEM		0.042	0.061	4.66	3.30	2.62	4.14	2.58	1.75
Ca/rP									
1.3		2.43^c^	2.32	51.0^a^	59.7^a^	71.4	68.7^a^	78.7	84.2
1.8		2.63^b^	2.20	47.6^b^	56.6^a^	66.4	60.9^b^	72.2	74.5
2.3		2.66^b^	2.07	35.5^c^	46.4^b^	54.5	51.9^c^	63.8	65.8
2.8		2.73^a^	1.94	27.4^d^	39.3^c^	47.9	44.9^d^	57.4	59.9
SEM		0.030	0.043	3.29	2.33	1.85	2.93	1.82	1.24
Phytase									
0		2.62	2.03	38.6	49.4	58.4	46.7	59.0	62.5
1000		2.60	2.23	42.1	51.6	61.7	66.5	77.1	79.7
SEM		0.021	0.030	2.33	1.65	1.31	2.07	1.30	0.87
*P*-value									
Ca/rP		<0.001	<0.001	<0.001	<0.001	<0.001	<0.001	<0.001	<0.001
Linear		<0.001	<0.001	<0.001	<0.001	<0.001	<0.001	<0.001	<0.001
Quadratic		0.001	0.959	0.317	0.237	0.540	0.866	0.966	0.038
Phytase		0.296	<0.001	0.133	0.195	0.014	<0.001	<0.001	<0.001
Ca/rP × Phytase		0.952	<0.001	0.159	0.124	0.023	0.207	0.021	0.001

a-g Values within a column without common superscript differ significantly (*P* ≤ 0.05).

1 Calculated rP content was 3.2, 2.5 and 2.2 g/kg in the starter, grower and finisher diets (CVB, 2018a), respectively, excluding the contribution of microbial phytase.

2 Nine replicate pens per treatment (n = 9).

A substantial increase in apparent P digestibility from the proximal to distal jejunum, with only a slight subsequent increase in the distal ileum (Table 5) was observed. Incremental dietary Ca/rP linearly reduced (*P* < 0.001) apparent P digestibility in all 3 intestinal segments. In the distal jejunum and distal ileum, a Ca/rP × phytase interaction on apparent P digestibility (*P*_interaction_ = 0.021 and 0.001, respectively) was observed. Increasing dietary Ca/rP from 1.3 to 2.8 reduced distal ileal P digestibility, which was more pronounced in broilers fed phytase free compared to phytase supplemented diets (29.5 vs. 19.1% units reduction). Incremental dietary Ca/rP linearly decreased serum P concentration, but this reduction was much smaller with phytase-supplemented diets than with phytase-free diets (*P*_interaction_ < 0.001).

### Duodenal mRNA Expression of Transporters and CLDN

Analysis of the broiler's duodenal mucosa on d 21 to 23 by RT-qPCR revealed that incremental dietary Ca/rP linearly reduced the mRNA expression levels of extracellular Ca^2+^ sensing receptor (**CaSR**), calbindin-D28k (**CaBP-D28k**) and plasma membrane Ca pump (**PMCA1**) by 70, 51, and 40%, respectively (*P* < 0.001, Table 6). Phytase had no impact on these Ca homeostasis-related genes. Transcript levels of apical Ca channel (**TRPV6**) were beyond the limit of detection and that of transient receptor potential canonical 1 (**TRPC1**), a nonspecific cation channel, were affected neither by dietary Ca/rP nor phytase treatment.

**Table 6:** Least square means of mRNA expression level of calcium (Ca) and phosphorus (P) transporters, vitamin D_3_ receptor (VDR) and claudin (CLDN) on d 21–23 in the broiler's duodenal mucosa in response to dietary Ca to retainable P ratio (Ca/rP) and microbial phytase intake^1^^,^^2^.

Ca/rP	Phytase, FTU/kg	Ca transporters				P transporters				VDR and CLDN			
		CaSR	TRPC1	CaBP-D28k	PMCA1	NaPi-IIb	PiT-1	PiT-2	XPR1	VDR	ZO-1	CLDN-2	CLDN-12
1.3	0	0.36	0.39	455	101	3.30	2.18^c^	392	5.32	2.75	1.44	23.0	0.23
1.8	0	0.25	0.38	349	80.5	3.53	3.60^a^	382	5.32	2.76	1.53	18.3	0.23
2.3	0	0.16	0.39	291	71.0	2.76	2.45^bc^	366	5.03	3.05	1.34	18.4	0.22
2.8	0	0.12	0.37	231	62.3	2.71	2.87abc	353	4.76	2.83	1.25	15.8	0.20
1.3	1,000	0.39	0.41	455	98.2	3.83	3.43^ab^	406	5.41	2.93	1.50	23.3	0.23
1.8	1,000	0.17	0.49	346	90.7	4.30	2.68abc	466	5.44	2.67	1.58	16.2	0.25
2.3	1,000	0.14	0.41	200	59.6	3.28	3.29^ab^	317	4.57	2.72	1.29	14.4	0.19
2.8	1,000	0.10	0.38	213	56.8	3.40	3.39^ab^	379	4.90	2.89	1.30	11.9	0.20
SEM		0.046	0.045	53.9	10.75	0.549	0.540	44.2	0.433	0.446	0.124	2.17	0.020
Ca/rP													
1.3		0.37^a^	0.40	455^a^	99.4^a^	3.57	2.81	399	5.37	2.84	1.47^ab^	23.2^a^	0.23^ab^
1.8		0.21^b^	0.44	347^b^	85.6^a^	3.91	3.14	424	5.38	2.71	1.55^a^	17.2^b^	0.24^a^
2.3		0.15^bc^	0.40	246^c^	65.3^b^	3.02	2.87	342	4.80	2.88	1.32^bc^	16.4^b^	0.21^bc^
2.8		0.11^c^	0.37	222^c^	59.5^b^	3.05	3.13	366	4.83	2.86	1.28^c^	13.8^c^	0.20^c^
SEM		0.033	0.032	38.1	7.60	0.388	0.382	31.3	0.307	0.315	0.088	1.54	0.014
Phytase													
0		0.22	0.38	332	78.6	3.07	2.77	373	5.11	2.85	1.39	18.9	0.22
1000		0.20	0.42	303	76.3	3.70	3.20	392	5.08	2.80	1.42	16.4	0.22
SEM		0.023	0.022	26.9	5.38	0.274	0.270	22.1	0.217	0.223	0.062	1.09	0.010
*P*-value													
Ca/rP		<0.001	0.104	<0.001	<0.001	0.071	0.746	0.053	0.097	0.952	0.008	<0.001	0.026
Linear		<0.001	0.275	<0.001	<0.001	0.052	0.562	0.071	0.028	0.826	0.005	<0.001	0.010
Quadratic		0.010	0.163	0.113	0.457	0.573	0.903	0.982	0.961	0.829	0.338	0.131	0.523
Phytase		0.412	0.271	0.287	0.671	0.026	0.119	0.398	0.912	0.841	0.662	0.029	0.863
Ca/rP × Phytase		0.454	0.385	0.571	0.542	0.986	0.035	0.218	0.727	0.873	0.927	0.460	0.519

Abbreviations: CaSR, Ca^2+^ sensing receptor; TRPC1, transient receptor potential canonical 1; CaBP-D28k, Calbindin D28k; PMCA1, plasma membrane Ca-ATPase; NaPi-IIb, sodium dependent phosphate transporter IIb; PiT-1, inorganic phosphate transporter 1; PiT-2, inorganic phosphate transporter 2; XPR1, polytropic retrovirus receptor 1; VDR, vitamin-D_3_ receptor; ZO-1, zonula occludens-1; CLDN, claudin.

Of note, mRNA expression of transient receptor potential cation channel subfamily V member 6 (TRPV6), sodium dependent phosphate transporter IIa (NaPi-IIa) and CLDN-16 were beyond limit of detection.

a-c Values within a column without common superscript differ significantly (*P* ≤ 0.05).

1 Calculated rP content was 3.2, 2.5 and 2.2 g/kg in the starter, grower and finisher diets, respectively, excluding the contribution of microbial phytase (CVB, 2018a).

2 Nine replicate pens per treatment (n = 9).

Duodenal expression of the apical sodium dependent P transporters, NaPi-IIb, PiT-2 and the candidate basolateral P extrusion channel, xenotropic and polytropic retrovirus receptor 1 (**XPR1**), were either significantly reduced or showed a trend with incremental dietary Ca/rP in a linear fashion (14, 8 and 23% reduction; *P* = 0.052, 0.071, and 0.028, respectively). Moreover, phytase increased NaPi-IIb mRNA by 20% (*P* = 0.026), but did not alter the levels of PiT-2 and XPR1 mRNA (*P* = 0.398 and 0.912, respectively). Expression of PiT-1 was affected by an interaction between Ca/rP and phytase (*P*_interaction_ = 0.035), with the expression being 57% higher with phytase in the lowest Ca/rP diet compared to the non-phytase group.

On the one hand, incremental dietary Ca/rP linearly decreased duodenal zonula occludens-1 (**ZO-1**), CLND-2, and -12 mRNA irrespective of phytase inclusion (13, 40, and 13% reduction, *P* = 0.005, < 0.001 and 0.010, respectively) while phytase inclusion, on the other hand, reduced CLDN-2 expression by 15% (*P* = 0.029). Neither dietary Ca/rP nor phytase affected the expression of vitamin D_3_ receptor (**VDR**) mRNA. Expression of CLDN-16 in the broiler's duodenal mucosa was beyond the limit of detection.

### Characteristics of Tibia Strength

Incremental dietary Ca/rP linearly and quadratically enhanced the stiffness of tibia in broilers, with a steeper slope in phytase-included than phytase-free diets (Table 7), as evidenced by the interaction effect (*P*_interaction_ = 0.001). Furthermore, the tibia breaking strength was linearly and quadratically increased (*P* < 0.001) with incremental Ca/rP or phytase inclusion, and tibia diameter tended to be quadratically increased with incremental Ca/rP reaching the maximal tibia diameter at Ca/rP of 1.8 (*P* = 0.075).

**Table 7:** Effects of dietary Ca to retainable P ratio (Ca/rP) and microbial phytase intake on the least square means of diameter and characteristics of breaking strength of tibia collected from broilers on d 21–23.^1^^,^^2^

Ca/rP	Phytase, FTU/kg	Diameter, mm	Stiffness, N/mm	Tibia breaking strength, N
1.3	0	6.0	57.0^d^	116
1.8	0	6.1	68.9^c^	139
2.3	0	6.2	77.1^b^	160
2.8	0	6.0	73.5^bc^	158
1.3	1,000	5.9	57.1^d^	122
1.8	1,000	6.2	81.0^b^	165
2.3	1,000	6.1	88.7^a^	178
2.8	1,000	6.0	95.0^a^	181
SEM		0.13	3.81	7.4
Ca/rP				
1.3		5.9	57.1	119^c^
1.8		6.2	75.0	152^b^
2.3		6.1	82.9	169^a^
2.8		6.0	84.2	169^a^
SEM		0.09	2.71	5.2
Phytase				
0		6.1	69.1	143
1,000		6.1	80.5	161
SEM		0.07	1.91	3.7
*P*-value				
Ca/rP		0.075	<0.001	<0.001
linear		0.392	<0.001	<0.001
quadratic		0.016	<0.001	<0.001
Phytase		0.706	<0.001	<0.001
Ca/rP × Phytase		0.591	0.001	0.242

a-d Values within a column without common superscript differ significantly (*P* ≤ 0.05).

1 Calculated rP content was 3.2, 2.5, and 2.2 g/kg in the starter, grower, and finisher diets, respectively, excluding the contribution of microbial phytase (CVB, 2018a).

2 Nine replicate pens per treatment (n = 9).

## DISCUSSION

A previous study by Hu et al. (2020) indicated that the reduction of dietary Ca content improves apparent P digestibility, but a too low dietary Ca content compromises growth performance and tibia breaking strength in broilers. In the present study, we hypothesized that microbial phytase supplementation improves dietary Ca digestibility, allowing a lower dietary Ca inclusion without compromising growth performance. This hypothesis, however, is not supported by our experimental results. We found that broilers fed with microbial phytase indeed displayed improved apparent distal ileal Ca digestibility, particularly in the lowest Ca/rP diet (Table 5). However, the additional absorbed Ca (0.32 g/kg diet) in the presence of phytase at the lowest Ca/rP diet was insufficient to alleviate the negative impact of a low Ca intake on growth performance, as indicated by a lower BWG and FI and higher FCR when birds received the lowest Ca/rP diet regardless of microbial phytase inclusion (Table 4). Furthermore, although mRNA expression of Ca absorption-related genes, including CaBP-D28k, PMCA1, CLDN-2 and -12 was highest in the duodenal mucosa for birds fed the lowest Ca/rP diet, this was independent of microbial phytase inclusion (Table 6). Therefore, our results did not support a reduction in dietary Ca inclusion in the presence of microbial phytase without compromising growth performance in broilers, which led us to reject the hypothesis.

### Dietary Ca and Microbial Phytase Interaction

Reducing dietary Ca content has been intensively studied since Ca can bind to IP which hampers the digestion of IP to lower IP esters, that is, IP6 to IP1 (Rodehutscord and Rosenfelder, 2016). It is demonstrated that microbial phytase inclusion enhances intestinal IP degradation (Walk and Rama Rao, 2020), thus it may also enhance dietary Ca digestibility. This assumption is supported by Li et al. (2020) who reported that microbial phytase inclusion (1,000 FTU/kg) significantly improved standardized ileal Ca digestibility (38 vs. 49%) in broilers, which supports our results (Table 5). Interestingly, dietary phytase inclusion increased apparent distal ileal Ca digestibility to a greater extent in the lowest Ca/rP diet, while phytase improved BWG and FI to a numerically greater extent in the higher Ca/rP diets (Table 4). This finding suggests that release of IP-Ca might not have been a major element for dietary phytase to augment the broiler's growth performance. Amerah et al. (2014) reported that microbial phytase inclusion improved BWG, FI, and FCR in broilers fed a Ca-sufficient, but not a Ca-deficient diet, which accompanied with an increased ileal digestibility of P, gross energy, and amino acids. They concluded that the positive impact of phytase on growth performance was attributed to a higher digestibility of other nutrients, such as amino acids, energy, and P rather than Ca. Thus, despite of a positive impact on Ca digestibility, dietary phytase inclusion would not restore the compromised growth performance of broilers when a Ca-deficient diet was offered.

Numerous studies have been published (e.g., Bello et al. (2019), Krieg et al. (2020), and Ajuwon et al. (2020)) demonstrating that microbial phytase inclusion improves IP degradation and P absorption in broilers. We also observed an improved apparent P digestibility in diets supplemented with microbial phytase. Noteworthy is the rather high distal ileal apparent P digestibility (i.e., 78.8%) without microbial phytase inclusion in the lowest Ca/rP diet (Table 5), indicating that broilers had a large potential to degrade IP and absorb IP-P upon low-Ca intake. Since the intrinsic phytase activity in the basal diet was low (Table 2), the high ileal P digestibility observed in the absence of exogenous phytase inclusion is probably attributed to the activity of endogenous epithelial and/or microbial phosphatases in the GIT of broilers. This idea is supported by Rodehutscord and Rosenfelder (2016), who in their literature survey found that approximately 70% of dietary IP6 is degraded in the distal ileum of broilers fed a low-Ca and low-P content diet devoid of phytase. Furthermore, we also observed a steep linear inhibitory effect on apparent ileal P digestibility with incremental dietary Ca/rP in the absence of phytase, indicating that the catalytic activity of endogenous epithelial and/or microbial phosphatases is significantly inhibited by dietary Ca. This is in line with the findings of Sommerfeld et al. (2018), who showed a strong inhibition of ileal IP degradation with dietary inclusion of mineral Ca and P in broilers fed phytase-free diets. To further clarify the source of endogenous phosphatase, these researchers conducted a similar study by feeding gamma-irradiated diets to gnotobiotic broilers (Sommerfeld et al., 2019). Since their data revealed a similar reduction of ileal IP degradation upon mineral Ca and P supplementation, it can be concluded that broilers have a great potential to absorb P from IP due to a high endogenous epithelial phosphatase activity, which can be inhibited by high levels of dietary Ca.

Dietary phytase inclusion alleviated the negative impact of dietary Ca/rP increment on apparent P digestibility in the distal jejunum and ileum (Table 5), which is largely in agreement with Sommerfeld et al. (2018), who reported that the negative impact of dietary supplementation of Ca and P on ileal IP degradation in broilers could be fully recovered by the inclusion of microbial phytase. As mentioned above, in the absence of microbial phytase, a high dietary Ca intake inhibited endogenous epithelial phosphatases activity and reduced distal ileal P digestibility. In contrast, in the presence of microbial phytase, the negative impact of a high dietary Ca intake on P digestibility was much smaller. It appears that the efficacy of the exogenous microbial phytase is less affected by dietary Ca inclusion compared to the endogenous epithelial phosphatases. An explanation for this difference is likely pH related, that exogenous microbial phytase is primarily active in the proventriculus and gizzard (Dersjant‐Li et al., 2015), while endogenous epithelial phosphatases are mostly active in the small intestine. The luminal pH in the small intestine is favorable to Ca-IP complexation, impeding the efficacy of endogenous epithelial phosphatases, while the low pH in the proventriculus and gizzard (approximately at pH 3), is unfavorable to Ca-IP complexation, making the efficacy of exogenous microbial phytase in the latter segments less sensitive to dietary Ca intake.

### Expression of Ca and P Related Transporters and Tight Junctions

We observed a downregulation of CaBP-D28k and PMCA1, CLDN-2 and -12 mRNA with incremental Ca/rP (Table 6) in the duodenal mucosa. These results are in line with the reduced apparent Ca digestibility at a higher Ca intake in the posterior small intestinal segments (Table 5), suggesting that both intestinal Ca transporters and CLDN are regulatable and contribute to whole body Ca homeostasis in broilers. The higher serum Ca level obtained with high Ca intake (Table 5) would activate the extracellular CaSR on the parathyroid gland, supressing parathyroid hormone (**PTH**) expression and secretion (Musgrove and Wolf, 2020). A reduced PTH circulation would reduce serum circulation of 1,25-dihydroxycholecalciferol (**1,25(OH)_2_D_3_**), thereby, downregulating expression of Ca transporters and CLDN in the GIT since the promoter of CaBP-D28k, PMCA1, CLDN-2 and -12 contains a VDR responsive element (Fujita et al., 2008; Proszkowiec-Weglarz and Angel, 2013). Another possible mechanism for the downregulation of Ca transporters might be that a high dietary Ca/rP increased luminal Ca content, which might activate extracellular CaSR, a transmembrane G protein-coupled receptor expressed in the GIT, thereby, directly reducing CaBP-D28k and PMCA1 expression, independent of circular 1,25(OH)_2_D_3_ (Lee et al., 2019; Chanpaisaeng et al., 2021).

Incremental Ca intake also led to the downregulation of duodenal P transporters NaPi-IIb, PiT-2, and XPR1 (Table 6), which is consistent with the lower apparent P digestibility in the jejunal and ileal part under these conditions (Table 5). Furthermore, the downregulation of P transporters was accompanied by a lower concentration of serum P, and dietary phytase inclusion increased serum P while upregulating duodenal expression of NaPi-IIb. Our observations are in line with the work of Huber et al. (2006), who demonstrated a positive correlation between plasma P and jejunal NaPi-IIb mRNA expression in laying hens. Expression of NaPi-IIb in the intestine of broilers might be under control of systemic hormones and regulators such as 1,25(OH)_2_D_3_ and fibroblast growth factor 23 (**FGF23**). It is conceivable that high Ca intake decreased serum 1,25(OH)_2_D_3_ (Hu et al., 2020), via the parathyroid-kidney axis, which in turn reduced intestinal NaPi-IIb mRNA expression. Furthermore, dietary phytase inclusion enhanced serum P concentration (Table 5), which might have stimulated FGF23 secretion from the bone (Ren et al., 2017). A higher serum FGF23 circulation has been shown to be coupled with a greater NaPi-IIb protein expression in the duodenal mucosa of layers (Ren et al., 2020). Taken together, expression of P transporters is consistent with intestinal P absorption in broilers.

### Site of Ca and P Absorption

It is generally accepted that Ca and P are primarily absorbed in the small intestine of chickens (Veum, 2010). In this study, we observed that an average of 95 and 85% of the prececal digestible P and Ca were absorbed proximal to the ileum, respectively indicating a limited contribution of the ileum to Ca and P absorption. Our previous study in broilers (Hu et al., 2020) demonstrated that approximately 85% of the distal ileal digestible P and Ca were absorbed anterior to the ileum, which is in line with results of the present study. Rodehutscord et al. (2012) compared apparent P digestibility in the proximal, medial, and distal ileum in broilers receiving diets with various P content. They found that P absorption was trivial in the ileum particularly in broilers fed diets with a P content above 5 g/kg. Their results are in line with the present study (P content 4.8 g/kg), and shows that the ileum plays a minor role in P absorption in broilers. Ileal Ca absorption has been less investigated but may not be substantial according to the present and our previous study (Hu et al., 2020).

Broilers need more Ca to optimize bone development than growth performance. As documented above, dietary Ca/rP quadratically increased growth performance with the maximum BWG in broilers fed a diet with Ca/rP of 1.8 (Table 4). However, tibia breaking strength and stiffness were compromised at Ca/rP of 1.8 as compared to higher ratios. Thus broilers need more Ca to optimize bone development than growth performance. This finding is in line with our previous study (Hu et al., 2020).

In conclusion, dietary phytase inclusion improves distal ileal apparent Ca digestibility but cannot recover the compromised growth performance in broilers fed a Ca-deficient diet. Incremental dietary Ca/rP reduces apparent Ca and P digestibility paralleled with a reduced duodenal expression of Ca and P transporters and CLDN, indicating that these transporters and CLDN contribute to the observed effect of Ca level and phytase on Ca and P absorption in chicken.
